# Tri-mannose grafting of chitosan nanocarriers remodels the macrophage response to bacterial infection

**DOI:** 10.1186/s12951-018-0439-x

**Published:** 2019-01-25

**Authors:** Juan Manuel Coya, Laura De Matteis, Alexandre Giraud-Gatineau, Anne Biton, Inés Serrano-Sevilla, Anne Danckaert, Marie-Agnès Dillies, Brigitte Gicquel, Jesus M. De la Fuente, Ludovic Tailleux

**Affiliations:** 1 Mycobacterial Genetics Unit, Institut Pasteur, Paris, France; 2 Instituto de Nanociencia de Aragon, Universidad de Zaragoza and CIBER-BBN, Saragossa, Spain; 3 CIBER-BBN, Instituto de Salud Carlos III, Madrid, Spain; 4 Unit for Integrated Mycobacterial Pathogenomics, CNRS, UMR 3525, Institut Pasteur, Paris, France; 5 Université Paris Diderot, Sorbonne Paris Cité, Cellule Pasteur, Rue du Dr. Roux, 75015 Paris, France; 6 Institut Pasteur – Bioinformatics and Biostatistics Hub – C3BI, USR 3756 IP CNRS, Paris, France; 7 Instituto de Ciencia de Materiales de Aragón (ICMA), CSIC-Universidad de Zaragoza, and CIBER-BBN, Edificio I+D, Calle Mariano Esquillor s/n, 50018 Saragossa, Spain; 8 UtechS Photonic BioImaging (Imagopole)-Citech, Institut Pasteur, Paris, France

**Keywords:** Chitosan nanocarriers, Surface grafting, Macrophages, *Mycobacterium tuberculosis*, Host response

## Abstract

**Background:**

Infectious diseases are still a leading cause of death and, with the emergence of drug resistance, pose a great threat to human health. New drugs and strategies are thus urgently needed to improve treatment efficacy and limit drug-associated side effects. Nanotechnology-based drug delivery systems are promising approaches, offering hope in the fight against drug resistant bacteria. However, how nanocarriers influence the response of innate immune cells to bacterial infection is mostly unknown.

**Results:**

Here, we used *Mycobacterium tuberculosis* as a model of bacterial infection to examine the impact of mannose functionalization of chitosan nanocarriers (CS-NCs) on the human macrophage response. Both ungrafted and grafted CS-NCs were similarly internalized by macrophages, via an actin cytoskeleton-dependent process. Although tri-mannose ligands did not modify the capacity of CS-NCs to escape lysosomal degradation, they profoundly remodeled the response of *M. tuberculosis*-infected macrophages. mRNA sequencing showed nearly 900 genes to be differentially expressed due to tri-mannose grafting. Unexpectedly, the set of modulated genes was enriched for pathways involved in cell metabolism, particularly oxidative phosphorylation and sugar metabolism.

**Conclusions:**

The ability to modulate cell metabolism by grafting ligands at the surface of nanoparticles may thus be a promising strategy to reprogram immune cells and improve the efficacy of encapsulated drugs.

**Electronic supplementary material:**

The online version of this article (10.1186/s12951-018-0439-x) contains supplementary material, which is available to authorized users.

## Background

Bacterial infection is a major cause of chronic infections and mortality. Tuberculosis (TB), which is caused by *Mycobacterium tuberculosis*, is the deadliest disease caused by a single infectious agent, ahead of HIV/AIDS and malaria. According to the most recent WHO report [1], there were 10.4 million TB cases in 2016 and the disease killed 1.7 million individuals. Even more serious is the worldwide emergence of drug-resistant bacteria, endangering the efficacy of antibiotics. Each year in the United States and Europe, 23,000 and 25,000 people, respectively, die as a direct result of antimicrobial resistance [2, 3]. The ability to cure multidrug resistant bacterial infections is more difficult, requiring treatment with more toxic and costly drugs, often with limited success. Hepatotoxicity, liver injury, skin reactions, and gastrointestinal and neurological disorders have frequently been observed as adverse effects. New strategies are thus urgently needed against resistant strains to shorten the duration of treatment and limit drug side-effects.

Nanoparticles are an attractive approach to increase the efficacy of antibiotics and decrease drug side-effects [4, 5]. Nanocarriers (NCs) are a broad family of submicron structures with unique size-dependent features, including high stability, efficient drug loading, controlled-release, and cell-targeting [6]. Previous studies have shown effective internalization of NCs by phagocytes, in particular macrophages (${\text{M}}{\upphi }{\text{s}}$), thus allowing delivering of antibiotics [7], antigens for vaccination [8], or contrast agents for biomedical imaging [9]. For example, liposomes and solid lipid nanoparticles have been shown to improve the activity of antibiotics, such as amikacin and vancomycin, against *Pseudomonas aeruginosa* and *Staphylococcus aureus* [10, 11]. Lipid NCs loaded with rifampicin, one of the first-line TB drugs, exhibit higher lung and ${\text{M}}{\upphi }$-specific targeting than the free drug in vivo [12, 13]. Polymeric nanoparticles composed of natural or synthetic polymers, such as chitosan and poly(lactide-*co*-glycolide) acid (PLGA), have shown good drug encapsulation and delivery [14–16]. Moreover, it was shown that nanoparticles are able to effectively target granuloma-like structures using transparent zebrafish embryos infected with *Mycobacterium marinum* as a model of TB infection, thus improving embryo survival and lowering bacterial load [7]. NCs have also been recently used to improve BCG-vaccine immunogenicity by enhancing the innate immune response to BCG vaccination [17].

Numerous NCs have been developed in the past decades using several experimental approaches. One of the best strategies to produce and stabilize NCs consists of using aqueous- or oil-core nanocapsules surrounded by a polymeric coating that can be synthetic, such as PLGA, or natural, such as chitosan. Chitosan coating provides several advantages, such as biocompatibility, biodegradability, and functional groups for biofunctionalization for cell targeting, making chitosan NCs (CS-NCs) an excellent delivery system. We previously reported the production of CS-NCs with improved stability, by nano-emulsion with a chitosan hydrogel coating, and their use as an efficient drug delivery system for the antimicrobial agent bedaquiline [18, 19]. Here, we performed an in-depth study of the interactions of CS-NCs with human monocyte-derived ${\text{M}}{\upphi }{\text{s}}$, resting or infected with *M. tuberculosis*, as a model of bacterial infection. mRNA sequencing allowed us to identify genes and pathways affected by CS-NC treatment. CS-NCs regulated the expression of relatively few genes but, surprisingly, the addition of tri-mannose carbohydrates to CS-NCs (CS-NCs-tri) profoundly remodeled the response of *M. tuberculosis*-infected cells.

## Results and discussion

### Chitosan NCs are efficiently internalized by ${\text{M}}{\upphi }{\text{s}}$ in an actin cytoskeleton-dependent manner

**Fig. 1:**
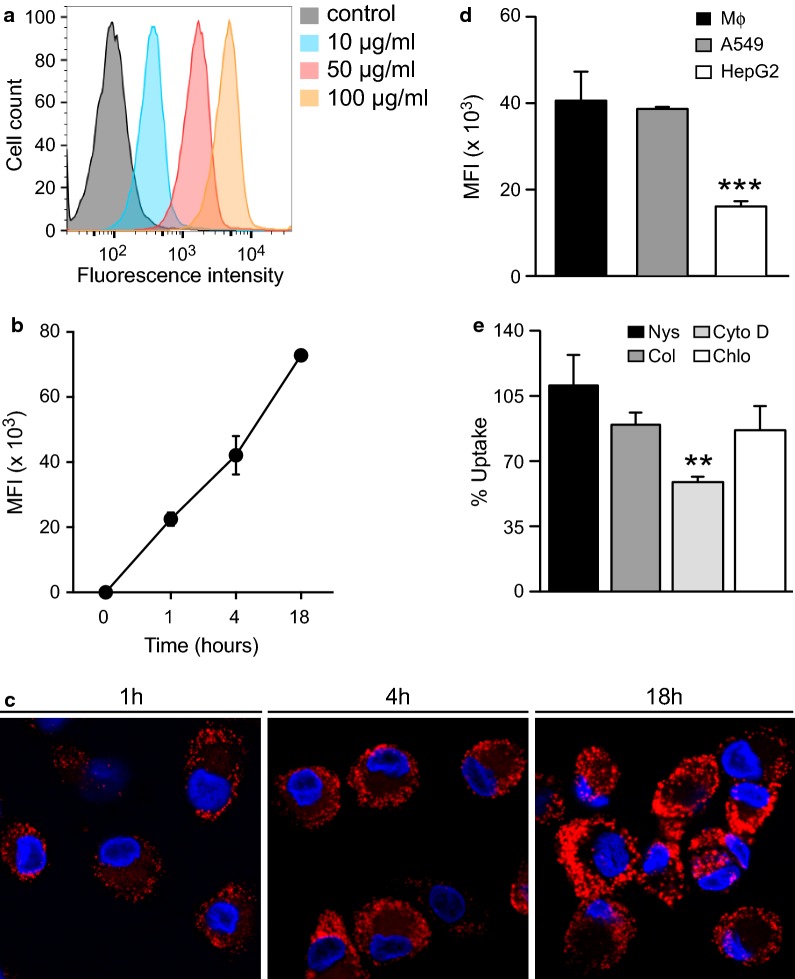
Cellular uptake of chitosan NCs. **a** ${\text{M}}{\upphi }{\text{s}}$ were incubated for 4 h with 10, 50, and 100 µg/ml Nile-Red-labelled CS-NCs. Particle internalization was assessed by FACS and the results expressed as the mean fluorescence intensity (MFI). **b** ${\text{M}}{\upphi }{\text{s}}$ were cultured for 1, 4, or 18 h in the presence of 100 µg/ml fluorescent CS-NCs and particle internalization was quantitatively assessed by FACS. **c** ${\text{M}}{\upphi }{\text{s}}$ were treated with 100 µg/ml fluorescent CS-NCs (red) for 1, 4, or 18 h and NC internalization visualized by confocal microscopy. DAPI (blue) was used to visualize nuclei. **d** 100 µg/ml Nile-Red-labelled CS-NC were incubated for 4 h with ${\text{M}}{\upphi }{\text{s}}$, A549 epithelial cells, or HepG2 hepatocytes. NP uptake was quantitatively analyzed by FACS. **e** ${\text{M}}{\upphi }{\text{s}}$ were incubated with 100 µg/ml fluorescent CS-NCs for 2 h with or without the pharmacological inhibitors nystatin, colchicine, cytochalasin D, or chlorpromazine. NC uptake was analyzed as previously described. Error bars represent the mean ± SD and significant differences between treatments are indicated by an asterisk, in which **p < 0.01 and ***p < 0.001

We first evaluated the capacity of human ${\text{M}}{\upphi }{\text{s}}$ to internalize CS-NCs. Monocyte-derived ${\text{M}}{\upphi }{\text{s}}$ were incubated for 4 h with Nile Red-labelled CS-NCs. After extensive washing, we quantitatively assessed particle internalization by FACS. ${\text{M}}{\upphi }{\text{s}}$ efficiently internalized CS-NCs in a dose- and time-dependent manner (Fig. 1a, b). The uptake of NCs was very fast. We observed Nile Red positive-${\text{M}}{\upphi }{\text{s}}$ in as little as 15 min (data not shown). We analyzed CS-NC-treated ${\text{M}}{\upphi }{\text{s}}$ by confocal microscopy after 1, 4, and 18 h of incubation to discriminate between binding of the NCs to the cell surface and their internalization. The NCs were localized intracellularly and accumulated with time (Fig. 1c). We next evaluated the capacity of other cell types, namely lung epithelial cells (A549) and hepatocytes (HepG2), to internalize CS-NCs. Hepatocytes internalized a smaller number of CS-NCs than ${\text{M}}{\upphi }{\text{s}}$ (0.6-fold less), whereas the uptake by epithelial cells was similar (Fig. 1d). These results confirm previous studies showing efficient internalization of CS-NCs by several cell types, including epithelial cells, hepatocytes, fibroblasts, and ${\text{M}}{\upphi }{\text{s}}$ [20].

We next treated ${\text{M}}{\upphi }{\text{s}}$ with inhibitors of phagocytosis, macropinocytosis, clathrin-mediated endocytosis, and caveolae-mediated endocytosis to decipher the molecular mechanisms involved in NC internalization [21]. Cytochalasin D inhibits phagocytosis by preventing actin polymerization [22]. Chlorpromazine reduces invagination via clathrin-mediated endocytosis by depleting the plasma membrane of clathrin and adaptor proteins and sequestering them on intracellular vesicles [23]. Nystatin interferes with caveolae-mediated endocytosis by increasing membrane fluidity via the depletion of cholesterol and reducing the formation of lipid caveolar rafts [24]. Colchicine decreases microtubule polymerization, thus inhibiting micropinocytosis [25]. Cells were incubated with fluorescent NCs for 2 h, with or without the inhibitors, and the internalization of NCs quantified by flow cytometry. Only cytochalasin D significantly decreased the uptake of CS-NCs (Fig. 1e). These results show that CS-NCs enter ${\text{M}}{\upphi }{\text{s}}$ via an actin cytoskeleton-dependent process, most likely phagocytosis.

### Chitosan NCs escape degradation in lysosomes

**Fig. 2:**
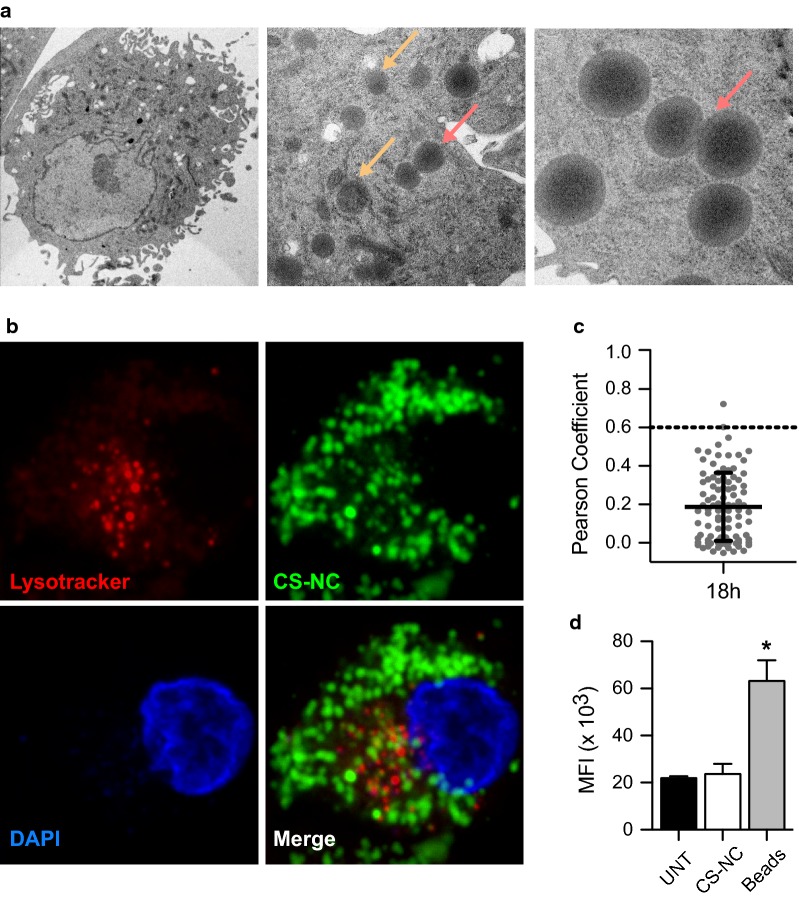
Intracellular localization of chitosan NCs. **a** ${\text{M}}{\upphi }{\text{s}}$ were cultured for 18 h in the presence of 100 µg/ml CS-NCs and intracellular visualization assessed by TEM. Yellow arrows: CS-NCs, Red arrows: NCs fusion. **b** ${\text{M}}{\upphi }{\text{s}}$ were incubated with 100 µg/ml DiD-labelled CS-NCs (green) for 18 h, co-stained with LysoTracker (red) and DAPI (blue), and visualized by confocal microscopy. **c** Pearson correlation coefficient between CS-NCs and acidic compartments (Lysotracker positive). Each dot represents one single cell (n = 98). Error bars represent the mean ± SD. **d** ${\text{M}}{\upphi }{\text{s}}$ were exposed to 100 µg/ml CS-NCs or latex beads for 18 h and incubated with Lysotracker Red. The intensity of the lysotracker staining was then quantified by FACS. Results are expressed as the mean fluorescence intensity (MFI). Significant differences between treatments and untreated controls are indicated by an asterisk, in which *p < 0.05. Error bars represent the mean ± SD

${\text{M}}{\upphi }{\text{s}}$ are professional phagocytes that are highly specialized to engulf and eliminate dead cells, cellular debris, and foreign particles, including bacteria and viruses [26]. We used transmission electron microscopy (TEM) and confocal microscopy to follow the fate of intracellular NCs inside this cell type. CS-NCs were easily detectable inside ${\text{M}}{\upphi }{\text{s}}$ after 1 h of incubation (data not shown). Surprisingly, the CS-NCs were still present after 18 h of incubation and were not degraded. Some even fused together, resulting in large NCs (Fig. 2a). This suggests that CS-NCs can resist lysosomal degradation and/or escape from the endosomal pathway. We could not detect membranes surrounding the CS-NCs, favoring the second hypothesis. We strengthened these results by incubating ${\text{M}}{\upphi }{\text{s}}$ with fluorescent CS-NCs and LysoTracker Red DND-99, a red fluorescent dye that stains acidic compartments, mainly lysosomes, in live cells, and analyzed their intracellular localization by confocal microscopy. Most CS-NCs did not colocalize with acidic compartments after 18 h of incubation (Fig. 2b, Pearson correlation coefficient < 0.6, Fig. 2c). Thus, we did not observe the formation of new lysosomal compartments upon treatment with CS-NCs. We quantified the intensity of the LysoTracker staining by flow cytometry. There was no difference between untreated cells and cells incubated with CS-NCs, whereas incubation with latex beads (used as a positive control) induced an increase of LysoTracker staining (Fig. 2d). Our results are consistent with those of previous studies. Indeed, it has been shown that polyethylenimine and chitosan can directly overcome lysosomal sequestration by membrane destabilization [27–29] or through a proton sponge effect [30–33]. NCs may enhance endosomal Cl-accumulation and osmotic swelling, leading to vesicle rupture.

### Effects of NCs on the ${\text{M}}{\upphi }$ response

**Fig. 3:**
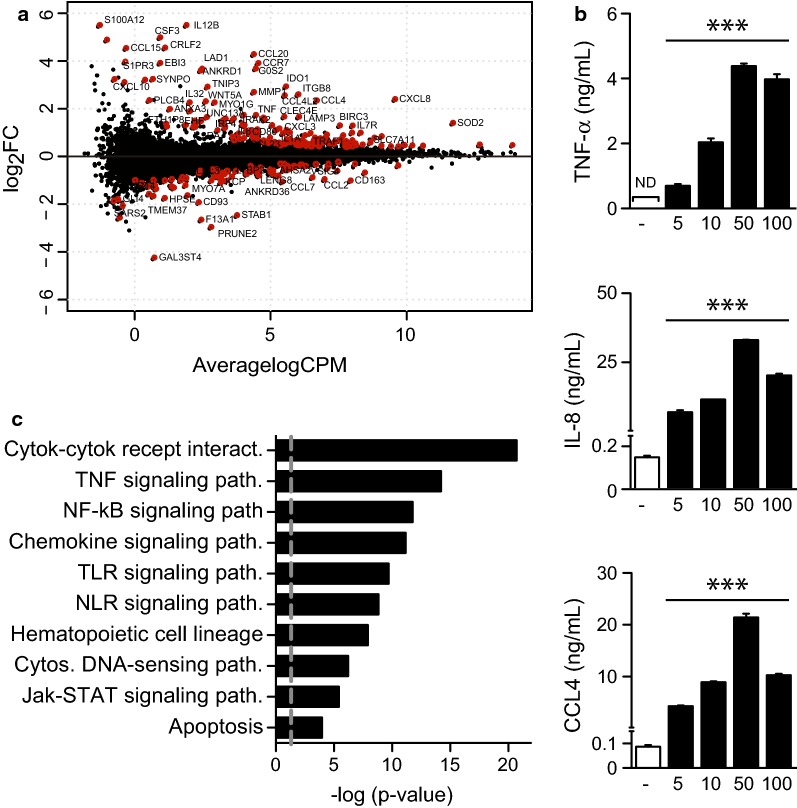
Biological processes regulated by chitosan NCs on ${\text{M}}{\upphi }{\text{s}}$. Transcriptome analysis was performed on ${\text{M}}{\upphi }{\text{s}}$ isolated from three different donors and treated with 10 µg/ml CS-NCs for 18 h. **a** MA plot showing differentially expressed (DE) genes by CS-NCs relative to untreated controls. Genes with an FDR < 0.05 are shown in red. **b** Validation of the expression of selected candidate genes by ELISA. One representative experiment (out of three) is shown. Error bars represent the mean ± SD and significant differences between treatments are indicated by an asterisk, in which ***p < 0.001. **c** Significantly enriched biological processes (KEGG analysis) for genes regulated by CS-NC treatment relative to untreated controls. *Interact.* interaction, *path.* pathway

The phagocytosis of particles by ${\text{M}}{\upphi }{\text{s}}$ modulates the expression of many genes, the number and extent depending on the receptors involved. In the context of NCs, it is important to decipher the impact of CS-NC internalization on the cell transcriptome to identify potential side effects. Indeed, it has been shown that NCs can regulate the expression of cell-cycle-related genes, modulate inflammation, and up-regulate the stress response [34–36]. We evaluated the host response to CS-NCs by comparing the transcriptional profiles of untreated ${\text{M}}{\upphi }{\text{s}}$ (control) with those incubated with CS-NCs. After 18 h of incubation, the cells were lysed and the mRNA sequenced. CS-NCs did not alter cell viability over an incubation period of 5 days (Additional file 1: Fig. S1). Detailed descriptions of our data processing, quality control analysis, and statistical modeling are available in the Methods section. We used a low-dose of NCs to mimic realistic doses of ${\text{M}}{\upphi }$ exposure in vivo and to avoid any response due to a high number of particles [37]. We identified 242 genes for which the expression was modulated by CS-NCs (FDR < 0.05, Fig. 3a and Additional file 2: Table S1). The expression of 156 genes was upregulated and that of 86 downregulated upon treatment. In particular, the expression of inflammation-related genes (IL-12B, IL-32, TNF-α and IL-6) and that of chemokines (CCL20, CCL4L2, CXCL8/IL-8, and CCL4) was strongly upregulated in the presence of chitosan. We examined a selected panel of genes to validate our transcriptomic data. We used ELISA to confirm the upregulation of TNF-α, interleukin 8 (CXCL8/IL-8), and CCL4 secretion in the supernatant of Μφs treated with various concentrations of NPs for 18 h (Fig. 3b).

We then classified the modulated genes on the basis of the annotation resources provided by KEGG [38] by performing gene-set enrichment analysis of the list of differentially expressed genes using the EnrichR tool [39]. The gene set differentially expressed by CS-NCs was significantly enriched for genes involved in cytokine–cytokine receptor interactions (p = 4.49E−20), TNF and NF-kB signaling (p = 1.59E−13 and 5.33E−11, respectively), chemokine signaling (p = 6.28E−11), and the TLR/NLR pathways (p = 2.86E−10 and 9.34E−10, respectively) (Fig. 3c and Additional file 3: Table S2). Chitosan is a naturally occurring polysaccharide derived from chitin, the second most common polysaccharide in nature. It is less common than chitin and can be found in certain species of fungi, but neither are expressed in mammals [40]. It is thus not surprising that this polysaccharide induces a pro-inflammatory response. Indeed, highly purified chitosan was shown to potently activate the NLRP3 inflammasome, increasing expression of the pro-inflammatory cytokine IL-1β [41]. CS-NCs induced expression of pro-inflammatory cytokines by human peripheral blood mononuclear cells, including IL-6, TNF-α and IFN-γ, among others [35]. In vivo, chitosan has been reported to induce an acute inflammatory response [42] and ${\text{M}}{\upphi }$ nitric oxide production and chemotaxis [43]. The ability of CS-NCs to promote inflammation could be useful when drug delivery is combined with immunotherapy to elicit innate and adaptive immune responses against pathogens [44] or tumor cells [45]. However, long-term inflammatory responses could be detrimental to the host. We thus decided to modify CS-NCs in order to decrease their pro-inflammatory properties, and we generated CS-NCs containing tri-mannose motifs. ${\text{M}}{\upphi }{\text{s}}$ express numerous lectins which can bind mannose, including the mannose receptor and DC-SIGN [46]. These receptors have immunomodulatory properties and can dampen inflammation upon ligand binding [47–49]. Targeting these receptors may thus be an attractive strategy to modulate the host response to CS-NCs.

### Fabrication and characterization of tri-mannose-chitosan NCs

**Fig. 4:**
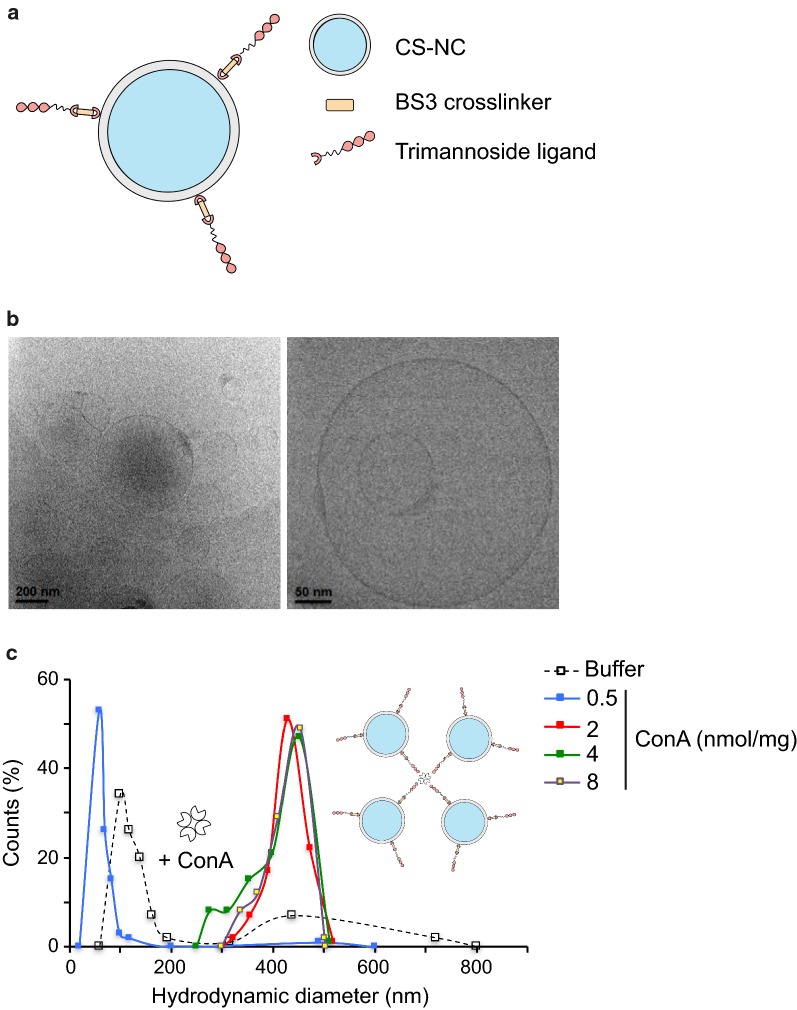
Morphological characterization of tri-mannose-grafted chitosan NCs. **a** Schematic representation of a mannosylated CS-NC. **b** CryoTEM image of CS-NCs-tri. **c** Hydrodynamic diameter of CS-NCs-tri incubated at various concentrations of concanavalin A, assessed by dynamic light scattering

The general procedure for ligand grafting on chitosan surfaces, using the homobifunctional crosslinker, bis(sulfosuccinimidyl) suberate (BS^3^), has been reported previously for other types of ligands [18]. Here, we adapted it for the linking of a tri-mannose ligand with an alkyl spacer chain and a primary amino group on the NC surface available for the reaction with the crosslinker (Fig. 4a and Additional file 4: Fig. S2a). We assessed the physicochemical properties of CS-NCs before and after grafting with tri-mannose. CS-NCs had a slightly higher mean diameter after the grafting process (Additional file 5: Table S3). The tri-mannose chain might strongly interact with the water of the medium, altering its structure and organizing the surrounding water molecules in a thicker hydration shell. In the case of Nile Red-labelled NCs, the non-grafted carriers were slightly less stable and their diameter appeared to be higher. After grafting, both labelled and non-labelled carriers had a hydrodynamic diameter of slightly less than 200 nm.

We next analyzed the morphology of the grafted NCs by cryo-electron microscopy. The NCs were homogeneous and the size of most of the spherical NCs present were in accordance with the dynamic light scattering analysis (Fig. 4b). The surface of the NCs showed no appreciable differences in morphology by electron microscopy after grafting. Nonetheless, we evaluated the outcome of the grafting process by Fourier transform infrared spectroscopy (FTIR) and by measuring the surface potential of NCs before and after grafting with the tri-mannose ligand (Additional file 4: Fig. S2b and Additional file 5: Table S3). Unmodified chitosan surfaces generally present high positive Z potential values in water and slightly acidic pH, due to the presence of protonated amino groups. After grafting with the tri-mannose ligand, the masking effect on the amino groups on the surface led to negative potential values. The high absolute values also indicated good colloidal stability of the samples under the measuring conditions.

Finally, we tested the surface availability of tri-mannose motifs using concanavalin A (ConA). ConA is a lectin that presents a tetrameric structure at physiological pH (7.4), formed by four identical subunits, each possessing a mannose-binding site. Because of this characteristic, ConA can be used as a bridge to produce a specific concentration-dependent, aggregate of grafted NCs that can be measured by DLS analysis. There was a slight stabilizing effect at low concentrations of the lectin, whereas there was a four-fold increase in diameter at concentrations above 2 nmol/mg_NC_ (Fig. 4c). The same experiment was carried out using non-grafted CS-NCs and there was no specific aggregation (Additional file 4: Fig. S2c). We observed a completely different behavior of non-grafted NCs in the presence of buffer only, showing, in this case, an important aggregation effect due to the presence of salt. Comparison of the results obtained with grafted and non-grafted NCs further demonstrate the efficacy of the grafting process on the chitosan surface and demonstrates the availability of the ligand for the affinity interaction with lectin receptors.

### Tri-mannose grafting dampens the response of ${\text{M}}{\upphi }{\text{s}}$ to CS-NCs

**Fig. 5:**
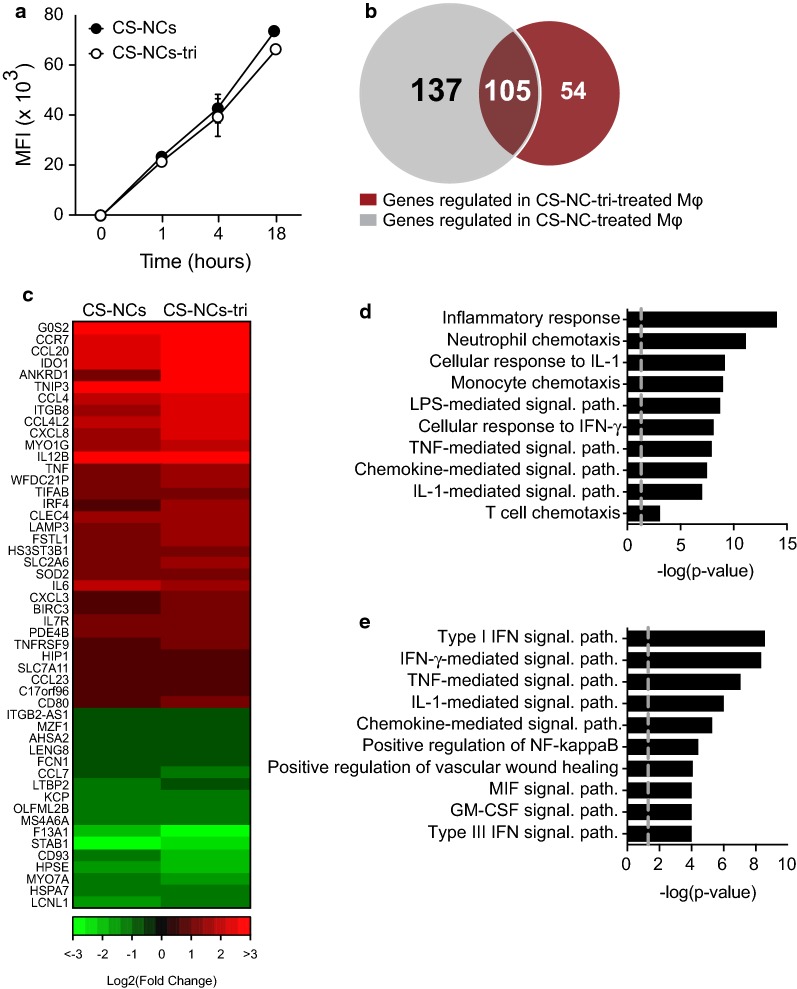
Schematic Differentially-expressed genes upon chitosan NC and tri-mannose-grafted chitosan NC treatment. **a** ${\text{M}}{\upphi }{\text{s}}$ were incubated with 100 µg/ml fluorescent CS-NCs and CS-NCs-tri. Internalization of the NCs was then assessed by FACS as in Fig. 1. **b**–**e** ${\text{M}}{\upphi }{\text{s}}$ from three individual donors were treated for 18 h with 10 µg/ml CS-NCs or CS-NCs-tri. The differentially-expressed genes were then identified by mRNAseq. **b** Venn diagram showing the number of genes regulated by NC treatment relative to untreated controls. **c** Genes induced by CS-NCs or CS-NCs-tri that displayed > 1.5-fold difference (p < 0.05) in expression were plotted in a heat map. **d** Pathway classifications provided by GO of the differentially-expressed genes upon treatment with CS-NCs and CS-NCs-tri. **e** Pathway classifications provided by GO of the genes differentially-expressed only upon treatment with ungrafted CS-NCs. *Sign. path*. signaling pathway

We first evaluated whether the addition of tri-mannose ligands to the surface of the NCs would improve their uptake by ${\text{M}}{\upphi }{\text{s}}$, as this cell type expresses several lectins at high levels, including the mannose receptor. There were no significant differences in internalization between NCs, with or without tri-mannose ligands (Fig. 5a). We obtained similar results with other cell types, such as A549 epithelial cells and HepG2 hepatocytes (Additional file 6: Fig. S3a). As for CS-NCs, the entry of CS-NCs grafted with tri-mannose motifs, hereafter designated CS-NCs-tri, was cytochalasin D-dependent, suggesting a phagocytic process (Additional file 6: Fig. S3b, Additional file 7: Fig. S4).

We next compared the ${\text{M}}{\upphi }$ response to CS-NCs and CS-NCs-tri. In resting ${\text{M}}{\upphi }{\text{s}}$, both nanocarriers modulated the expression of relatively few genes relative to other stimuli, such as LPS, in which more than 2500 genes have been described to be differentially expressed (Figs. 3a, 5b) [50]. We identified only 242 and 159 genes differentially expressed by cells upon treatment with respectively CS-NCs and CS-NCs-tri, of which 105 were in common (Fig. 5b and Additional file 8: Table S4). The expression of 42% of the genes differentially expressed upon treatment with CS-NCs-tri was upregulated. We first focused our analysis on the genes in common between the two nanocarriers. Unfortunately, the grafting of tri-mannose failed to decrease the pro-inflammatory properties of chitosan. The expression of the potent inflammatory mediators, TNF-α and IL-6, and several chemokines reported to attract immune effector cells (monocytes, neutrophils, dendritic cells, and activated T cells) was upregulated in the presence of both nanoparticles (Fig. 5c). Modulated genes were then classified based on the annotation resources provided by GeneOntology. The set of genes upregulated by NCs was significantly enriched for genes involved in immune activation, such as inflammatory response (p = 9.65E−15), neutrophil chemotaxis (p = 7.79E−1), and the cellular response to IL-1 (p = 7.48E−10) (Fig. 5d and Additional file 9: Table S5). The shared response between CS-NCs and CS-NCs-tri most likely reflects the recognition of chitosan by ${\text{M}}{\upphi }{\text{s}}$ as a pathogen-associated molecular pattern (PAMP), as described above.

We then evaluated the impact of tri-mannose grafting on the ${\text{M}}{\upphi }$ response by analyzing the differentially expressed genes specific of each nanocarrier. Functional classification of the 137 genes differentially regulated by only ungrafted CS-NCs showed that the type I-interferon (IFN-I) signaling pathway was the most significantly upregulated (Fig. 5e and Additional file 10: Table S6). This pathway comprised IL1A, SOCS3, CSF3, RSAD2, OAS2, MX1, IFI6, IRF, GP1BA, and EREG. Type I-IFNs play a key role in the antiviral response and are also involved in autoimmunity (lupus and genetically based interferonopathies) [51], cancer [52], and the immune escape mechanisms of bacterial pathogens, such as *Mycobacterium tuberculosis* [53]. Indeed, blood transcriptional profiling in TB patients, non-human primates, and mice infected with *M. tuberculosis* have shown up-regulation of type I-IFN response related genes, and type I-IFN was associated with impaired control of bacterial growth and elevated pulmonary immunopathology in murine models of tuberculosis [54]. Similar results were obtained with other bacteria, such as *Listeria monocytogenes*, *Brucella abortus*, and *Staphylococcus aureus* [55, 56]. In the context of bacterial infections, CS-NCs-tri may offer the advantage of boosting the immune response while avoiding prolonged IFN-I signaling, which leads to immune dysfunction and bacterial escape. Note that functional classification of the 54 genes regulated by only CS-NCs-tri did not permit to identify significant pathways, probably because of too few differentially-expressed genes.

### Tri-mannose modulates the response of *M. tuberculosis*-infected ${\text{M}}{\upphi }{\text{s}}$

**Fig. 6:**
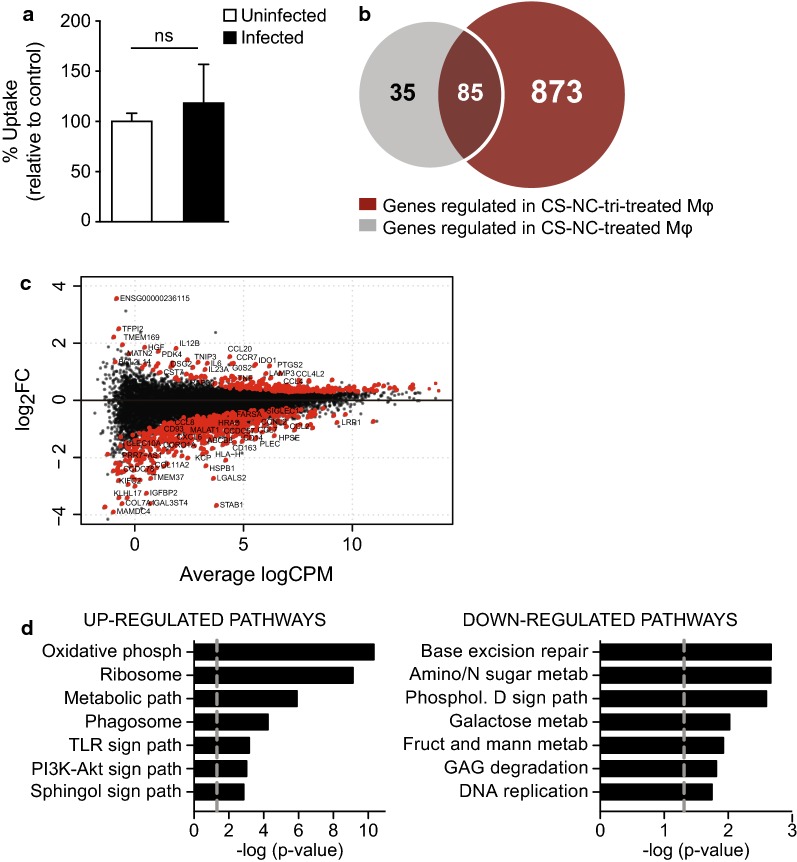
Grafting of tri-mmanose motifs on chitosan NCs modulates the response of *M. tuberculosis*-infected ${\text{M}}{\upphi }{\text{s}}$. **a** ${\text{M}}{\upphi }{\text{s}}$ were infected, or not, with *M. tuberculosis* and then treated with 100 µg/ml fluorescent CS-NC-tri for 18 h. Internalization of the CS-NC-tri was then assessed by FACS and the results expressed as the percentage uptake relative to uninfected cells treated with fluorescent CS-NC-tri (controls). The results are presented as the mean ± SD of three independent experiments. **b** Venn diagram showing differentially-expressed genes by *M. tuberculosis*-infected ${\text{M}}{\upphi }{\text{s}}$ treated with CS-NCs or with CS-NCs-tri, as in Fig. 5b. **c** MA plot showing differentially expressed genes following CS-NC-tri treatment relative to untreated infected controls. Genes with an FDR < 0.05 are shown in red. **d** Graph of significantly enriched biological processes (KEGG analysis) for genes up-regulated (left graph) or down-regulated (right graph) by CS-NC-tri treatment in *M. tuberculosis*-infected ${\text{M}}{\upphi }{\text{s}}$. The gray line indicates − log of p = 0.05. *met.* metabolism, *phosph*, phosphorylation, *sign. path.* signaling pathway

Bacterial infection induces important remodeling of the infected cell transcriptome [57]. Nanoparticles may thus affect uninfected and infected cells in a different manner. We evaluated the impact of the two types of NCs on the response of bacteria-infected ${\text{M}}{\upphi }{\text{s}}$. We used a similar approach as described above. Briefly, ${\text{M}}{\upphi }{\text{s}}$ were infected with a virulent strain of *M. tuberculosis* and then treated with CS-NCs or CS-NCs-tri. After 18 h of incubation, the cell transcriptome was analyzed by mRNA sequencing. There was no difference in the uptake of NCs between naïve and infected cells (Fig. 6a). Unexpectedly, the treatment of *M. tuberculosis*-infected ${\text{M}}{\upphi }{\text{s}}$ with CS-NCs-tri induced significant remodeling of the cell transcriptome. Overall, 958 genes were differentially expressed in cells incubated with CS-NCs-tri, whereas only 120 were differentially expressed in CS-NCs treated cells (Fig. 6b and Additional file 11: Table S7). Among the 958 genes, 873 were specific to CS-NCs-tri and the expression of most were downregulated (67%, Fig. 6c). The set of genes specifically modulated by CS-NCs-tri was enriched for genes involved in oxidative phosphorylation (up-regulation, p = 4.73E−11), metabolic pathways (p = 1.23E−06), and sugar metabolism (downregulation, p = 2.16E−3) (Fig. 6d and Additional file 12: Table S8). These results suggest that tri-mannose grafting affect the mitochondrial machinery and remodel cellular metabolism. Metabolic signaling dictates the fates and functions of many cell types, including T lymphocytes, B cells, and ${\text{M}}{\upphi }{\text{s}}$. For example, ${\text{M}}{\upphi }{\text{s}}$ can be schematically classified into two main classes, depending on their activation status: inflammatory or M1 ${\text{M}}{\upphi }{\text{s}}$, and alternatively activated M2 ${\text{M}}{\upphi }{\text{s}}$, involved in wound healing and angiogenesis. It has been shown that M2 polarization is dependent on fatty acid oxidation and oxidative phosphorylation [58, 59]. Tri-mannose ligands may thus favor M2 polarization in the context of bacterial infection.

## Conclusions

Here we dissected in detail the response of human ${\text{M}}{\upphi }{\text{s}}$ to two chitosan-based NCs, containing tri-mannose motifs at their surface or not. We identified a core response to both NCs, mainly associated with innate immune cell activation. We also detected unique pathways to CS-NCs and CS-NCs-tri. Some pathways, such as that of type I-IFN, have been shown to be exploited by certain pathogens to escape the immune response. It would thus be very informative to evaluate whether such NCs can hinder such pathogen strategies. Our study also showed that grafting NCs with tri-mannose can remodel the transcriptome of *M. tuberculosis*-infected cells, in particular affecting the regulation of many metabolic pathways. Further experiments will allow gaining insights into the mechanisms and consequences of cell metabolism modulation by tri-mannose grafting. The possibility to modulate cell metabolism by grafting ligands to the surface of the nanoparticles offers new perspectives. Indeed, many diseases, including infectious diseases, are associated with metabolic dysfunction. The grafting of nanoparticles with metabolic modulators in adjunction to conventional drugs may thus be a promising strategy to treat such diseases.

## Methods

### Materials

All reagents were purchased from Sigma-Aldrich unless otherwise specified. Tween^®^ 20 and absolute EtOH were purchased from Panreac Química S.L.U. Bis(sulfosuccinimidyl) suberate (BS^3^) was purchased from Pierce Biotechnology Inc. and 4-aminobutyl 2-*O*-(a-d-mannopyranosyl) 2-*O*-(a-d-mannopyranosyl) a-d-mannopyranoside was purchased from Omicron Biochemicals Inc.

### Macrophages and cell lines

Blood mononuclear cells were isolated by Ficoll-Paque centrifugation (GE Healthcare Life Sciences). CD14^+^ monocytes were isolated by positive selection using CD14 microbeads (Miltenyi Biotec) and allowed to differentiate into ${\text{M}}{\upphi }{\text{s}}$ in the presence of RPMI 1640 supplemented with 10% fetal bovine serum, 2 mM Glutamine and granulocyte–macrophage colony-stimulating factor (GM-CSF) (20 ng/ml; R&D Systems) over a 6-day period (hereafter defined as cell culture medium). Cell cultures were fed every 2 days. Human lung epithelium A549 cells (Sigma) were cultured in MEM. Human Hep G2 hepatocytes (Sigma) were cultured in EMEM (EBSS) supplemented with 10% fetal bovine serum, 2 mM Glutamine and 1% non-essential amino acids. Cultures were incubated at 37 °C in 5% CO_2_.

### CS-NC synthesis

CS-NCs were prepared as previously described [18]. For fluorescently labelled NCs, 100 μg Nile Red fluorophore was added to 40 ml organic phase before adding this solution to the aqueous phase for nanoemulsion formation. After 15 min, chitosan was added to stabilize the nanoemulsion. Finally, the chitosan-coated nanoemulsion was added to 200 ml 50 mM Na_2_SO_4_. Capsules were separated from Na_2_SO_4_ by ultracentrifugation (30 min, 69,673 × G, 10 °C), washed with 100 mL water, centrifuged again, and resuspended in water. The endotoxin concentration was < 0.05 EU.

### Flow cytometry

CS-NCs were labeled by encapsulation of Nile Red. ${\text{M}}{\upphi }{\text{s}}$, epithelial cells, and hepatocytes (4 × 10^5^ cells/ml) were grown on 24-well plates for 24 h in cell culture medium, followed by 1 to 18 h of treatment with various concentrations of fluorescent NCs. After incubation, cells were extensively washed with PBS to remove extracellular NCs, harvested, and resuspended in 4% paraformaldehyde for analysis using a CytoFLEX flow cytometer (Beckman Coulter). When indicated, cells were preincubated for 1 h with inhibitors in serum-free RPMI medium. The media was then changed to cell culture medium containing inhibitors plus fluorescent particles (100 μg/ml) and further incubated for 2 h. NC-only treated cells were used as positive controls and compared to inhibitor plus NC-treated cells. Concentrations were obtained from the literature for chlorpromazine (10 μg/ml), colchicine (2 μg/ml), cytochalasin D (10 μg/ml), and nystatin (20 μg/ml). All inhibitors were obtained from Sigma-Aldrich. More than 10,000 events per sample were recorded. The analysis was performed using FlowJo software.

### Confocal microscopy

${\text{M}}{\upphi }{\text{s}}$ (4 × 10^5^ cells/ml) were grown on 12-mm circular coverslips in 24-well tissue culture plates for 24 h in cell culture medium, followed by 1, 4 and 18 h of treatment with 100 μg/ml fluorescent NCs. Cells were then extensively washed with PBS and subsequently stained when indicated for 1 h with 1 mM LysoTracker^®^ Red DND-99 (ThermoFisher Scientific), following the manufacturer’s protocol. Cells were then washed twice with PBS, fixed with 4% paraformaldehyde for 30 min at RT, and mounted on a glass slide using Fluoromount mounting medium containing 1 µg/ml 4′,6-diamidino-2-phenylindole (DAPI) (Invitrogen). Cells were analyzed with a Leica TCS SP2 Confocal System. Z-stack optical sections were acquired at 0.3-µm-depth increments. Deconvolution and alignment of complete image stacks was performed with Huygens Pro (version 14.10, Scientific Volume Imaging). Each cell was analyzed with “Colocalization Analyzer” module to get quantitative information about the amount of spatial overlap between NCs and LysoTracker^®^ in data channels.

### Transmission electron microscopy (TEM)

The intracellular localization of CS-NCs in ${\text{M}}{\upphi }{\text{s}}$ was assessed by TEM. Cells incubated with 100 μg/ml NCs were fixed with 4% glutaraldehyde in 0.2 M sodium cacodylate pH 7 for 2 h at 4 °C. The cells were then washed twice with PBS and resuspended in 1 ml of 0.1% glutaraldehyde. Resin blocks were cut into 50-nm slices using an ultramicrotome. Samples were observed in a FEI Tecnai T20 microscope operating at 200 kV.

### Cytotoxicity assay

${\text{M}}{\upphi }{\text{s}}$ (0.1 × 10^6^ cells/well) were grown in 96-well plates for 24 h, followed by treatment with various concentrations of NCs for the indicated times. Cell viability was determined by MTT assay following the manufacturer’s protocol (Trevigen).

### RNA isolation, library preparation, and sequencing

Total RNA from macrophages was extracted using QIAzol reagent (Life Technologies) and purified over RNeasy columns (Qiagen). The quality of all samples was assessed with an Agilent 2100 bioanalyzer (Agilent Technologies) to verify RNA integrity. Only samples with a good RNA yield and no RNA degradation (ratio of 28S to 18S, > 1.7; RNA integrity number > 9) were used for further experiments. cDNA libraries were prepared with the Illumina TruSeq RNA Sample Preparation Kit v2 and were sequenced on an llumina HiSeq 2500 at the CHU Sainte-Justine Integrated Centre for Pediatric Clinical Genomics (Montreal, Canada). STAR v2.5.0b [60] was used to map RNA-seq reads to the hg38 reference genome and quantify gene expression (option-quantMode GeneCounts) by counting the fragments overlapping the Ensembl genes (GRCh38 v. 83). Differential expression analysis was performed using a generalized linear model with the R Bioconductor package edgeR v3.16.5 [61] on the genes with more than one count per million (CPM) in at least two samples. The model formula used in edgeR (~ Donor + Infection + Infection:Donor + Infection:Treatment + Donor:Treatment) contained: the main effects for Donor and Infection, interactions of Donor with Infection and Treatment to adjust for various responses to infection and treatment between donors, and a nested interaction of Infection with Treatment because we were interested in the infection-status-specific treatment effects. The latter was used to extract differentially expressed genes between NC-treated and untreated samples under the infected and uninfected conditions.

### Mycobacteria and ${\text{M}}{\upphi }$ infection

*Mycobacterium tuberculosis* H37Rv was grown from a frozen stock to mid-log phase in 7H9 medium (Becton–Dickinson), supplemented with albumin–dextrose–catalase (ADC, Difco), and ${\text{M}}{\upphi }$ infection carried out as previously described [62]. Before infection, bacteria were washed three times and re-suspended in 1 ml PBS. Clumps were dissociated by 30 passages through a needle and then allowed to sediment for 5 min. The density of bacteria in the supernatant was verified by measuring the OD_600_ and aliquot volumes defined to allow one bacterium-per two cell infections. Cells were infected in 24-well plates with each well containing 0.5 × 10^6^ cells in 1 ml medium containing GM-CSF (R&D Systems). After 2 h of incubation at 37 °C, infected cells were washed three times in PBS to remove extracellular bacteria and incubated in fresh medium. *M. tuberculosis* strain H37Rv, expressing green-fluorescent protein (GFP) (GFP-*M. tuberculosis*), carried the pEGFP plasmid (gift from G. Stewart, Imperial College, London, U.K.), which encodes resistance to hygromycin and harbors the *gfp* gene under the control of the mycobacterial P*hsp60* constitutive promoter.

### Enzyme-linked immunosorbent assay (ELISA)

At 18 h after NC treatment, supernatants from treated and untreated macrophages were filtered (pore size, 0.22 μm; Millipore). Levels of TNF-α, IL-8, and CCL4 (R&D) were determined in triplicate by ELISA following the protocol of the assay kit manufacturers.

### Tri-mannose-CS-NC synthesis and characterization

NCs (20 mg) were diluted in 10 mM borate buffer pH 8.2 for grafting with tri-mannose ligand. First, 50 nmol BS^3^ crosslinker was added for each mg of NCs and the reaction incubated for 30 min under stirring at room temperature. Then, a fourfold excess of 4-aminobutyl 2-*O*-(a-d-mannopyranosyl) 2-*O*-(a-d-mannopyranosyl) a-d-mannopyranoside, corresponding to a total amount of 4 μmoles of tri-mannose ligand, was added and the reaction mixture incubated for 2 h under stirring at 40 **°**C. Finally, an excess of 50 mM TRIS–HCl buffer pH 8.2 was added to quench any unreacted linker. Grafted NCs were washed three times, including centrifugation for 1 h at 16,000 rpm and 4 **°**C, to separate them from residual reactants. The NC concentration in a water suspension was obtained by measuring the weight of a fixed volume of sample after freeze-drying. The hydrodynamic diameter and polydispersity index (PDI) of the NCs were measured by dynamic light scattering analysis using a Brookhaven 90Plus DLS instrument and the Photo-Correlation Spectroscopy (PCS) technique. The endotoxin concentration was < 0.05 EU.

### Cryogenic transmission electron microscopy

Specimens were vitrified in liquid ethane and analyzed in a TEM microscope at low temperature. The vitrification process was performed in an FEI Vitrobot: a 3-µl drop of an aqueous suspension of the material was placed on a TEM Quantifoil carbon grid, excess water blotted away at the Vitrobot with filter paper, and the grid freeze-plunged into liquid ethane. Samples were then transferred under a liquid nitrogen atmosphere to a Gatan TEM cryo-holder equipped with a liquid nitrogen reservoir. TEM images were obtained in a Tecnai T20 (FEI), operated at 200 kV, coupled to a Veleta CCD camera.

### Determination of the surface potential

The surface potential was measured using a Plus Particle Size Analyzer (Brookhaven Instruments Corporation). NCs were analyzed in a 1 mM KCl suspension at a concentration of 0.01 mg/ml of material.

### Fourier transform infrared spectroscopy analysis

A JASCO FT/IR—4100 Fourier transform infrared spectrometer in a frequency range of 600–4000 cm^−1^ was used to carry out the analysis using a resolution of 2 cm^−1^ and a scanning number of 32.

### Concanavalin A aggregation test

NCs were diluted in 10 mM TRIS–HCl buffer pH 7.4 at a concentration of 0.2 mg/ml and various amounts of concanavalin A (0.5 to 8 nmol/mg_NC_) added. The mixture was left for 120 min for the interaction to occur and the degree of aggregation was subsequently measured by determining the hydrodynamic diameter of the NCs.

### Statistical analysis

Means were analyzed by 1-way ANOVA or the unpaired two tailed Student’s *t* test, for which p values < 0.05 (*), < 0.01 (**), and < 0.001 (***) were considered to be statistically significant. Analyses were performed using the Prism 5 program for MAC OS X (GradhPad Software). *p*-values for the RNA sequencing data were adjusted for multiple comparisons using the Benjamini–Hochberg method, producing an adjusted *p*-value or false-discovery rate (FDR). An FDR < 0.05 was considered to be statistically significant.

## Additional files

**Additional file 1: Fig. S1.** Cytotoxicity of chitosan NCs. M$\upphi$s were exposed to 100 µg/ml CS-NC for 18 h and 5 days. Cell viability was measured by the MTT assay.

**Additional file 2: Table S1.** Differentially-expressed genes by M$\upphi$s upon chitosan NC treatment. FDR < 0.05.

**Additional file 3: Table S2.** KEGG enrichment of differentially expressed genes upon chitosan NC treatment. p-value < 0.05.

**Additional file 4: Fig. S2.** Physicochemical and biological properties of tri-mannose-grafted chitosan NCs. a Chemical structure of the chitosan, BS3 linker, and trimannoside used to perform the grafted NCs. b FTIR analysis of CS-NCs before and after grafting with tri-mannose ligands. c Hydrodynamic diameter of non-grafted CS-NCs incubated with various concentrations of concanavalin A.

**Additional file 5: Table S3.** Dynamic light scattering and Z-potential analysis. NCs were characterized in terms of size (hydrodynamic diameter), polydispersity index (PDI), and surface potential (Z-potential).

**Additional file 6: Fig. S3.** Cellular uptake of tri-mannose-grafted chitosan NCs. a 100 µg/ml of Nile-Red-labelled CS-NCs-tri were incubated for 4 h with M$\upphi$s, A549 epithelial cells, or HepG2 hepatocytes. NP uptake was analyzed by FACS as mentioned above. b M$\upphi$s were incubated with 100 µg/ml fluorescent NCs for 2 h with or without nystatin, colchicine, cytochalasin D, or chlorpromazine. NC uptake was analyzed by FACS.

**Additional file 7: Fig. S4.** TEM micrographs of internalized tri-mannose-grafted chitosan NCs. M$\upphi$s were cultured for 1 a or 18 h. b in the presence of 100 µg/ml CS-NCs-tri. Intracellular localization was then assessed by TEM. Yellow arrows: CS-NCs, Red arrows: NCs fusion. Note that NC fusion leads to the formation of big nanoparticles in some cells at 18 h post-treatment.

**Additional file 8: Table S4.** Differentially expressed genes by M$\upphi$s upon tri-mannose-grafted chitosan NC treatment. First table: all genes differentially expressed. Second table: genes modulated by both NCs. Third table: genes modulated only by CS-NCs-tri. FDR < 0.05.

**Additional file 9: Table S5.** GO enrichment of genes differentially expressed by both chitosan NCs and tri-mannose-grafted chitosan NCs. p-value < 0.05.

**Additional file 10: Table S6.** GO enrichment of genes differentially expressed only upon treatment with chitosan NCs. p-value < 0.05.

**Additional file 11: Table S7.** Genes differentially expressed by *M. tuberculosis* infected M$\upphi$s upon treatment with NCs. First table: genes modulated by CS-NCs. Second table: genes modulated by CS-NCs-tri. FDR < 0.05.

**Additional file 12: Table S8.** KEGG enrichment of genes differentially expressed by *M. tuberculosis* infected M$\upphi$s upon treatment with tri-mannose-grafted chitosan NCs. First table: up-regulated processes. Second table: down-regulated processes. p-value < 0.05.

## Abbreviations

ConA: concanavalin A
CS-NCs: chitosan nanocarriers
FTIR: Fourier transform infrared spectroscopy
M$${\upphi }$$: macrophage
NCs: nanocarriers
PLGA: poly(lactide-*co*-glycolide) acid
TB: tuberculosis
TEM: transmission electron microscopy
WHO: World Health Organization
