# Supplementary figures and images for: Tri-mannose grafting of chitosan nanocarriers remodels the macrophage response to bacterial infection
Source: J Nanobiotechnology. 2019 Jan 25;17:15. doi: 10.1186/s12951-018-0439-x (PMC6346558; doi:10.1186/s12951-018-0439-x)

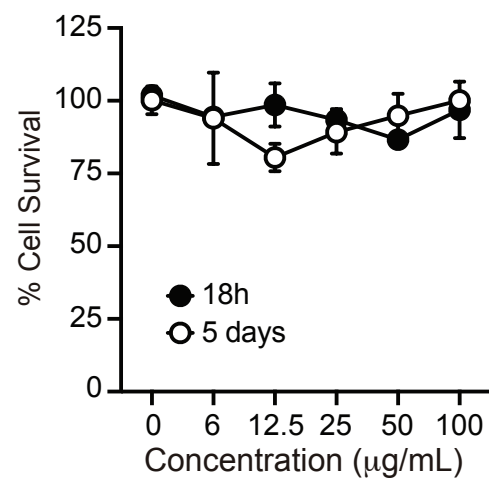

Supplement: Supplementary file 1 — Additional file 1: Fig. S1. Cytotoxicity of chitosan NCs. M$\upphi$ϕs were exposed to 100 µg/ml CS-NC for 18 h and 5 days. Cell viability was measured by the MTT assay. [file 12951_2018_439_MOESM1_ESM.pdf]

a

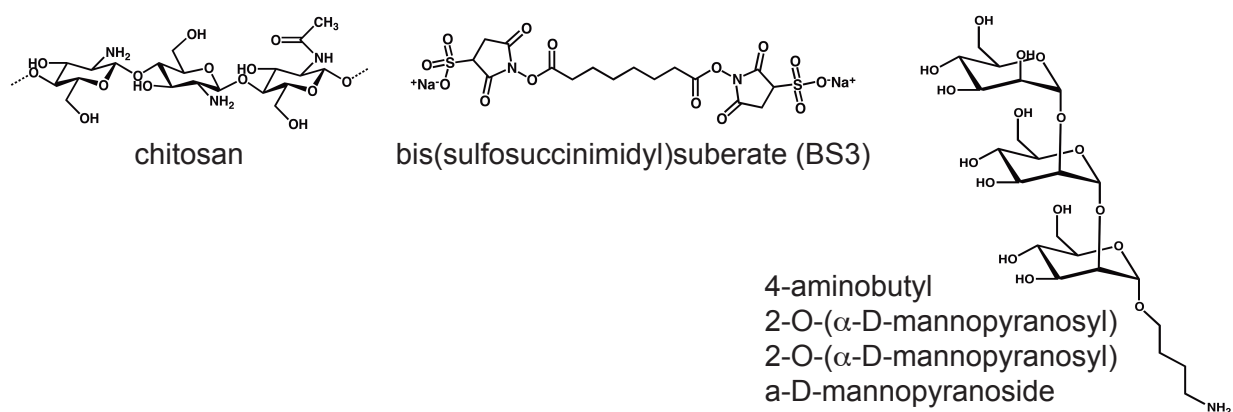

b

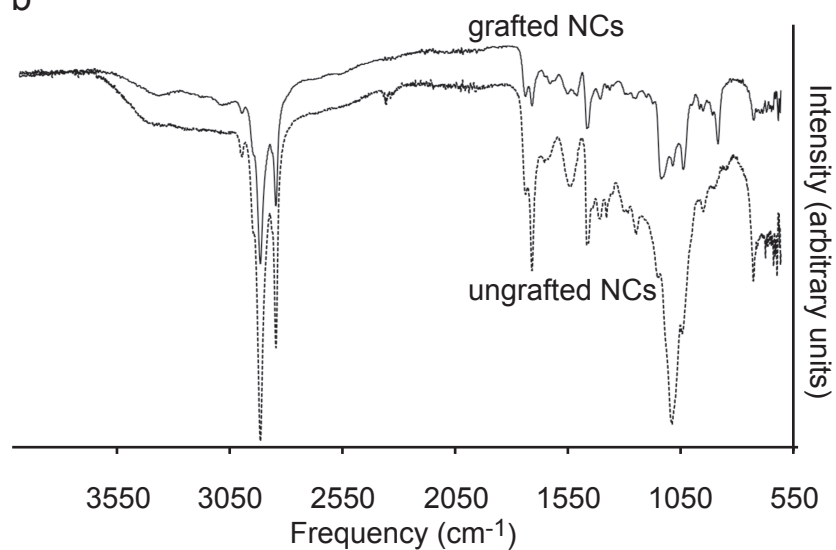

c

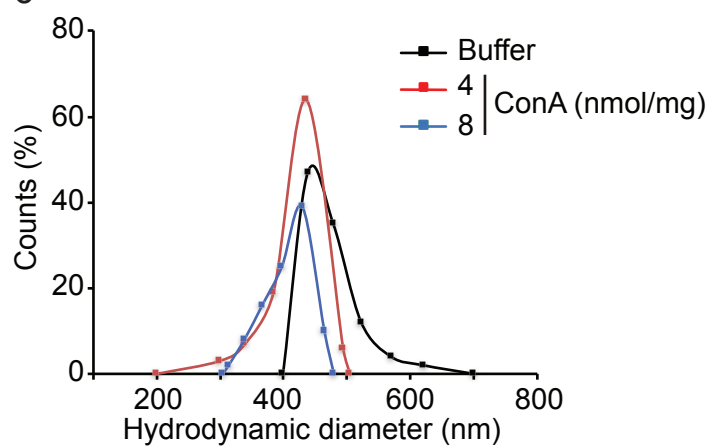

Supplement: Supplementary file 4 — Additional file 4: Fig. S2. Physicochemical and biological properties of tri-mannose-grafted chitosan NCs. a Chemical structure of the chitosan, BS3 linker, and trimannoside used to perform the grafted NCs. b FTIR analysis of CS-NCs before and after grafting with tri-mannose ligands. c Hydrodynamic diameter of non-grafted CS-NCs incubated with various concentrations of concanavalin A. [file 12951_2018_439_MOESM4_ESM.pdf]

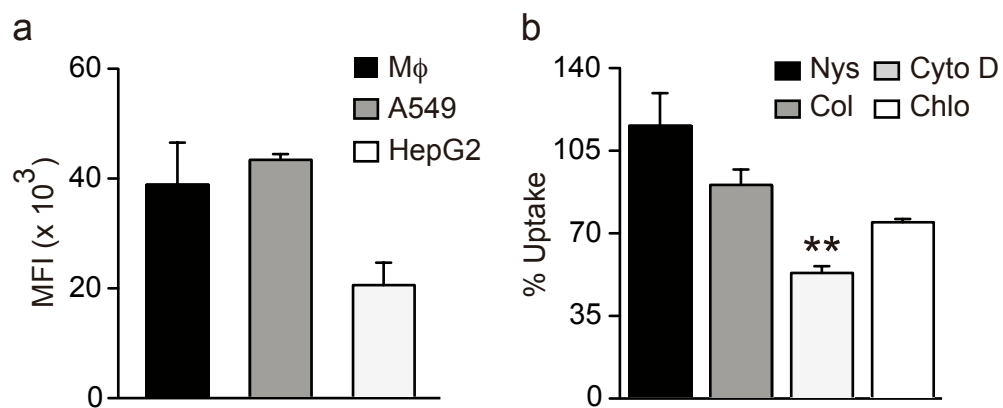

Supplement: Supplementary file 6 — Additional file 6: Fig. S3. Cellular uptake of tri-mannose-grafted chitosan NCs. a 100 µg/ml of Nile-Red-labelled CS-NCs-tri were incubated for 4 h with M$\upphi$ϕs, A549 epithelial cells, or HepG2 hepatocytes. NP uptake was analyzed by FACS as mentioned above. b M$\upphi$ϕs were incubated with 100 µg/ml fluorescent NCs for 2 h with or without nystatin, colchicine, cytochalasin D, or chlorpromazine. NC uptake was analyzed by FACS. [file 12951_2018_439_MOESM6_ESM.pdf]

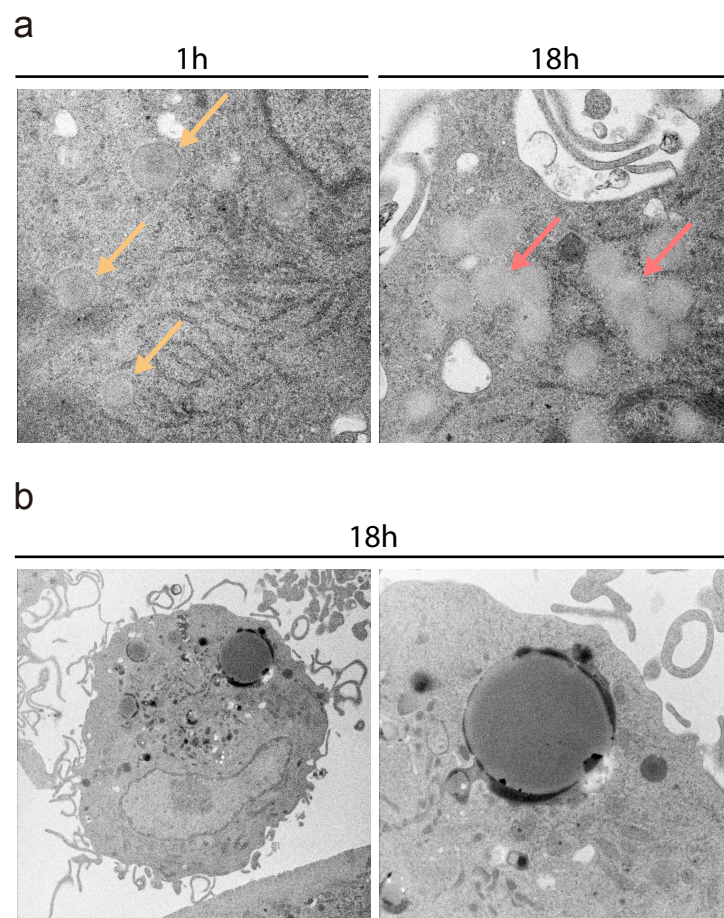

Supplement: Supplementary file 7 — Additional file 7: Fig. S4. TEM micrographs of internalized tri-mannose-grafted chitosan NCs. M$\upphi$ϕs were cultured for 1 a or 18 h. b in the presence of 100 µg/ml CS-NCs-tri. Intracellular localization was then assessed by TEM. Yellow arrows: CS-NCs, Red arrows: NCs fusion. Note that NC fusion leads to the formation of big nanoparticles in some cells at 18 h post-treatment. [file 12951_2018_439_MOESM7_ESM.pdf]
