# Supplementary material for: Tri-mannose grafting of chitosan nanocarriers remodels the macrophage response to bacterial infection
Source: J Nanobiotechnology. 2019 Jan 25;17:15. doi: 10.1186/s12951-018-0439-x (PMC6346558; doi:10.1186/s12951-018-0439-x)
Supplement: Supplementary file 5 — Additional file 5: Table S3. Dynamic light scattering and Z-potential analysis. NCs were characterized in terms of size (hydrodynamic diameter), polydispersity index (PDI), and surface potential (Z-potential). [file 12951_2018_439_MOESM5_ESM.pdf]

Nanocapsule	Hydrodynamic diameter (nm)	PDI	Z-Potential
CS-NCs	103.3	0.214	+21.19
CS-NCs-Tri	180.2	0.235	-23.81
Fluorescent CS-NCs	194.5	0.259	+29.9
Fluorescent CS-NCs-tri	182.1	0.233	-36.09

Coya_Additional table 3
